# Raman mapping on Ag-covered nanostructured substrates as an innovative approach for rapid estimation of post-mortem interval on human whole blood

**DOI:** 10.1007/s00414-026-03882-2

**Published:** 2026-06-26

**Authors:** Sara Sablone, Giuseppe Strisciullo, Andrea Nicola Cardinale, Nicola Antonio Colabufo, Maximilian Ries, Annalisa Convertino, Maurizio Pagliara, Andrea Starace, Valentina Mussi

**Affiliations:** 1 Section of Legal Medicine, Interdisciplinary Department of Medicine, University of Bari Aldo Moro, Bari Policlinico Hospital, Bari, Italy; 2 Section of Legal Medicine, Forensic Toxicology Laboratory, Interdisciplinary Department of Medicine, University of Bari Aldo Moro, Bari Policlinico Hospital, Bari, Italy; 3 Department of Pharmacy-Pharmaceutical Sciences, University of Bari, 70125 Bari, BA Italy; 4 Thermo Fisher Scientific GmbH, Im Steingrund 4-6, 63303 Dreieich, Germany; 5 Institute for Microelectronics and Microsystems, National Research Council (IMM-CNR), Via del Fosso del Cavaliere 100, 00133 Rome, Italy; 6 Levanchimica Srl, Viale Tommaso Columbo 41, 70132 Bari, Italy; 7 Thermo Fisher Scientific Spa, Via San Bovio, 3, 20054 Segrate, MI Italy

**Keywords:** Post-mortem interval, Raman spectroscopy, Surface Enhanced Raman Scattering, Nanowires, Human whole blood, Hypoxanthine

## Abstract

The estimation of the early post-mortem interval (PMI) in human corpses remains a challenging goal for forensic sciences. By exploiting label-free Raman mapping on non-treated human whole blood directly deposited on innovative nanostructured Surface Enhanced Raman Scattering (SERS) substrates, we propose a novel approach to rapidly obtain information on the presence of different levels of hypoxanthine (Hx) as a post-mortem biochemical marker (BPM). The efficacy of the method was tested on several whole blood samples from human corpses, spanning a time window between 3 and 32 h after death. The methodology, which allows the identification of metabolic and endogenous sources of Hx concentration increase, can be further engineered to develop dedicated automatable devices and holds potential for on-field applications during medical and forensic site inspections and investigations.

**Supplementary Information:**

The online version contains supplementary material available at https://doi.org/10.1007/s00414-026-03882-2.

## Introduction

Among the most important tasks of forensic sciences and criminal investigations there is the estimation of the post-mortem interval (PMI). Traditionally, it is based on the observation of corpse physical modifications in terms of body temperature, hypostasis, and muscle stiffness [1], with a prevailing reliability of the body temperature decrease assessment [2]. This procedure, although satisfying Daubert standards [3] and meeting the criteria for scientific evidence admissibility in the courts, rest on an empirical model by Marshall and Hoare [4], describing a set of external and internal conditions for body, such as body weight and surface area, fat and muscle representation, clothing, environmental temperature, microclimate, and so on [2, 5–8]. Nevertheless, this method is reliable only for relatively short PMIs (i.e., several hours since death or until the corpse temperature is leveled with the environmental one) and may be affected by intrinsic variables such as antemortem hyper- or hypothermia, while other traditional methods, such as isotope methods, DNA degradation, and forensic entomology [9–12], are useful for longer or very longer PMIs (weeks and years). In association with traditional methods, immunohistochemical (IHC) techniques also offer additional information (especially within the first two weeks after death) by enabling a more accurate estimation of PMI [13, 14].

Latest approaches to PMI estimation rely on the analysis of post-mortem biochemical markers (BPMs) changes, within the context of so-called post-mortem biochemistry (or thanatochemistry) in univariate analysis, or metabolomics in multivariate analysis. As these novel methods may expand the limits of time-after-death estimation, they can improve the accuracy and reliability of PMI assessment if combined with the traditional temperature-based nomogram use. The biochemical analyses include the measurements of macromolecule degradation (such as nucleic acids and proteins) and the monitoring of post-mortem changes in the body fluids' metabolomic composition [15–19].

The application of these novel methods to forensic studies on PMI evaluation requires to establish which tissue is the most suitable for analysis, and which compounds are the most promising for the specific inquiry. Undoubtedly, the optimal PMI biomarkers would be the metabolites whose body fluid levels change always with the same pattern (even better if linearly) for a relatively long time after death.

Metabolomic and biochemical analysis of post-mortem changes have been performed on plasma and blood serum [20–27], vitreous humor (VH) and aqueous humor (AH) [24–32], cerebrospinal, pericardial, and synovial fluids [33–37], suggesting that such compounds (e.g., sodium, potassium, chloride, ammonia, phosphate, lactate, hypoxanthine, choline, glycerol, taurine, succinate, urea, uric acid, and creatinine) might have a role as BPMs.

Notably, hypoxanthine (Hx) is produced during the intermediate stages of purine catabolism in humans, being a degradation product of adenosine triphosphate (ATP) [38]. Antemortem, under physiological conditions and with adequate levels of available oxygen, Hx is oxidized by xanthine oxidase (located in the liver and mucosa of the small intestine of mammals) and converted into uric acid, which is the final excreted product of human purine metabolism. Therefore, in the bloodstream, the end product of the purine catabolism pathway is Hx due to the absence of the enzyme xanthine oxidase in blood and vessels. In the context of tissue hypoxia, the metabolism of xanthine and uric acid from Hx is reduced or blocked [39–42] because of the following mechanisms: an increase in adenosine monophosphate (AMP), a decreased conversion of Hx into uric acid, and an inhibition of the xanthine oxidase enzyme [38, 43–47].

Thus, Hx is a valuable candidate as a BPM and it is expected that its concentration should increase after death due to the disruption of energy-dependent purine catabolism in oxygen-deficient biological matrices [24, 48].

Most of the results reported in literature, mainly obtained for animals owing to practical difficulties of collecting post-mortem human samples, demonstrate a stronger Hx’s correlation with PMI and a lesser influence by external factors (such as the ambient temperature) than other BPMs, also for time windows longer than 24 h [48]. Moreover, except for bones and pericardial fluid, which are the only human biological matrices where a time-dependent decline in Hx concentration was detected (with the former allowing the widest Hx-related PMI estimations, until over 20 years after death), the relevant scientific literature confirms a Hx increasing-related PMI prediction reliability in all biofluids (up to 100 h) by regression models with contained standard deviations [48]. This general Hx behaviour has been observed both in plasma/serum and VH — as well as in AH and cerebrospinal fluid (CSF) — over time after death (up to 120 h in VH), with anatomically isolated biological matrices (mostly, the VH) granting more reliable PMI estimations because of their slower autolytic changes and preservation from external physical-biochemical conditions [48–52].

However, detailed studies have not been carried out [22] and, except for VH [48], the possible linearity of the Hx-revealed trend is not fully and widely demonstrated and substantiated [22, 28].

Moreover, the biochemical methods employed to quantify Hx, such as high performance liquid chromatography (HPLC), nuclear magnetic resonance (NMR), and mass spectrometry (MS), are rather complex, expensive, and time consuming, or require sample preparation and specific fluorescence labelling, like enzymatic assays.

Here we propose a completely different and novel approach, exploiting label-free Raman mapping on human corpses’ whole blood directly deposited on innovative nanostructured substrates, to rapidly obtain information on the presence of different levels of Hx as a BPM.

Raman spectroscopy is a technique that explores molecular vibrational modes by shining intense monochromatic light onto a sample: the majority of the excitation photons are elastically scattered, maintaining the same energy as the incident ones, but a small fraction undergoes inelastic scattering revealing physical–chemical information about the target. When applied to biological samples, this technique enables the non-destructive and label-free analysis of complex organic materials, making it an invaluable tool in the field of bioanalytics and diagnostics.

However, Raman spectroscopy has some limitations due to the reduced efficiency of the inelastic process, leading to quite low signal levels. As a consequence, it requires a high-power excitation which could damage biological samples. For this reason, some signal amplification strategies have been developed based on the exploitation of plasmon resonances of nanometric metallic structures placed in close proximity to the studied molecules, named Surface-Enhanced Raman Spectroscopy (SERS). Several different SERS approaches have been proposed over time, making use of dispersed metal nanoparticles [53, 54] or nanostructured metal substrates.

We recently developed an innovative SERS platform based on a disordered array of silver-coated silicon nanowires (Ag/SiNWs) with notable advantages associated to reduced fabrication costs, no need for precise tuning of the size of nanostructures, and simplicity of the direct drop casting methodology for sample analysis [55]. Ag/SiNWs have been successfully applied to rapidly analyse different targets, such as DNA [56, 57], cells [58, 59], and glycated albumin [60]. The significant SERS effects arise from synergistic mechanisms: hotspot formation, i.e., regions of intense electromagnetic near field localization between neighboring metal-covered NWs, and simultaneous molecule trapping within these hotspots [60]. In the present study, the platform was exploited to study Hx in whole blood samples collected from human corpses, thus highlighting a peculiar correlation of the relative Raman signal with the PMI and allowing for investigation of the time window between very few hours (1–2) and several hours after death (18–23).

To the best of our knowledge, this study represents the first application of Raman spectroscopy for estimating early PMIs directly from whole blood. Although further validation is needed, we envision that the presented methodology has the potential to support and advance the traditional PMI estimation approaches in forensic practice.

## Materials and methods

### Sample collection

Whole blood samples were collected from human corpses undergoing forensic and medico-legal investigations ordered by judicial authorities over a three-year research period. The donor cadavers, 15 in total, were selected based on the time since death knowledge (such as in cases of death witnessed or documented in medical records) and only before the body storage in a refrigerator, to explore the natural time-related modification of BPMs. Overall, the known PMIs spanned the time window between 3 and 32 h after death*.* The causes of death, better defined after the required medico-legal investigations, were both natural and violent. Table 1 summarizes the main anagraphic and thanatological characteristics of the selected cases.

**Table 1:** Main anagraphic and thanatological characteristics of the selected cases

	**SEX**	**AGE** **(y.o.)**	**CAUSE OF DEATH**		**PMI (hours)**
			***NON-VIOLENT CAUSE***	***VIOLENT CAUSE***	
**Donor 1**	M	21		polytrauma by road accident	3
**Donor 2**	F	84	natural causes		3.5
**Donor 3**	F	43		acute drug poisoning	4
**Donor 4**	M	72		head trauma	6
**Donor 5**	M	45		encephalic head trauma with progressive coma and cardio-circulatory arrest	9
**Donor 6**	M	67	natural causes		9.5
**Donor 7**	M	39		acute drug poisoning	13
**Donor 8**	M	68	natural causes		14.5
**Donor 9**	M	57	spontaneous subarachnoid hemorrhage with irreversible coma		15.7
**Donor 10**	M	45		polytrauma by road accident	16.2
**Donor 11**	M	20		polytrauma by road accident	18
**Donor 12**	M	35		asphyxiation by hanging	20
**Donor 13**	F	43		acute drug poisoning	24
**Donor 14**	M	20		polytrauma by road accident	32.3
**Donor 15**	M	28		polytrauma by road accident	45

Ex vivo blood sampling was performed by drawing the biofluid with a needle-equipped syringe from the corpse's large inguinal vessels. About 1 ml of the blood sample was slowly dispensed in a plastic tube, which was closed with a screw cap and labeled to identify the donor cadaver, the time of death, and the time of sampling (to calculate the PMI). Then, the fresh whole blood samples (without pre-chemical treatments or anticoagulants) were stored at − 18^◦^C. The samples were defrosted immediately before being drop-casted on the SERS measurement substrates, i.e., characterized by Raman mapping "as obtained".

### SERS substrate fabrication and characterization

**Fig. 1:**
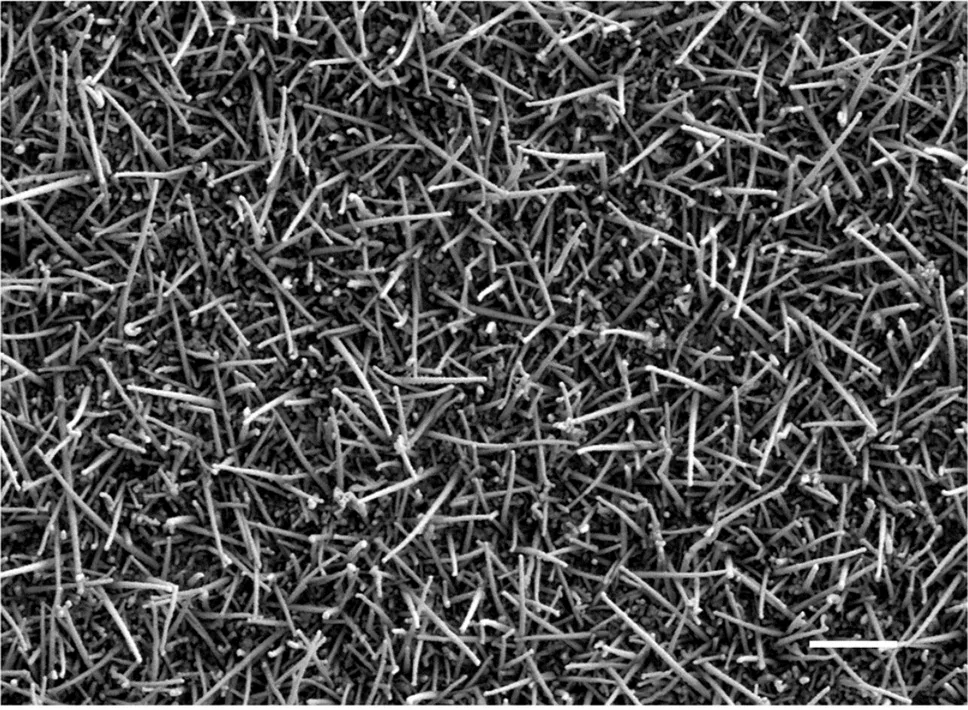
SEM image of a disordered array of Ag/SiNWs used as SERS substrate. Scale bar 1 $\mu \mathrm{m}$

Firstly, SiNWs were fabricated by plasma-enhanced chemical vapor deposition (PECVD) on Si wafers. Nanowire growth was obtained via vapor–liquid-solid mechanism by first evaporating a 2 nm-thick Au film onto the substrate acting as catalyst. SiNWs growth was performed using SiH4 and H2 as precursors at a total pressure of 1 Torr and flow ratio SiH4/(H2 + SiH4) fixed to 1:10. The substrate temperature was kept at 350 °C and plasma was ignited using 13.6 MHz radiofrequency at fixed power of 5 W. The resulting SiNWs array was then coated with a 100 nm-thick Ag film by evaporation to obtain the final Ag/SiNW SERS platform (more details on the growth procedure can be found in [50]). The morphology of the Ag/SiNWs was observed by a field emission scanning electron Microscope (SEM, ZEISS Sigma 300) at an accelerating voltage of 5 kV. The size of the NWs was determined by combined measurements from top and cross section views (see Fig. 1 showing a dense ensemble of disordered, randomly oriented Ag/SiNWs around 2–4 μm long, with an average bottom diameter of about 60–80 nm, and a cylindrical shape).

### Raman analysis

3 µl of each liquid whole blood sample were deposited on the Ag/SiNWs platform and left to dry for an hour at room temperature (RT). Then, Raman maps were acquired on the border of the drop in a back-scattering configuration through a DXR2xi Thermo Fisher Scientific Raman-Imaging Microscope. The measurements were performed by exciting the samples at 532 nm with 2 mW laser power and a 50 × long focal distance objective with a numerical aperture of 0.5, leading to an estimated power density in the laser focus of about 6 mW/µm^2^. High resolution spectra (1800 lines/mm diffraction grating) were acquired with a spectral resolution of about 2 cm^−1^ in the range 50–1800 cm^−1^. Each point spectrum of the map resulted from 10 accumulations of 10 ms acquisitions and the maps were collected over a square area with a fixed step-size of 2 μm for a total of about 3000 spectra. The spectra calculated as the average over the maps were compared after smoothing (Savitzky-Golay filter with a 5-point window), fluorescence background subtraction (polynomial of order 3), and baseline correction. Band intensities were calculated by a Lorentzian fit. The ratio between the intensities of the Raman bands at about 724 and 750 cm^−1^, named R, was used as a PMI marker.

## Results and discussion

Figure 1 shows a SEM image of the SERS substrate used for signal collection. As reported in previous works [56, 61], target solutions are drop casted on the top of such NWs’mats, and the Raman signal is acquired after drop drying. During evaporation, the NWs physically trap target molecules, while hotspots (i.e., regions of intense electromagnetic near field localization) form in the narrow gaps between neighboring metal-covered NWs, providing an effective platform for SERS sensing [56, 60, 61].

In this case, when whole blood drops were deposited on the surface of the Ag/SiNW substrates, an additional drying effect was observed: the liquid component (serum) accumulated at the border of the dried drop, while the solid component (red blood cells, white blood cells, and platelets) remained at the center. In other words, the substrate naturally induced a partial centrifugation effect on the sample, allowing for the separate measurement of different elements of the complex whole blood target. This differential distribution is likely related to the distinct physicochemical properties of the blood components, which affect their interactions with the nanostructured substrate and their mobility during drying.

**Fig. 2:**
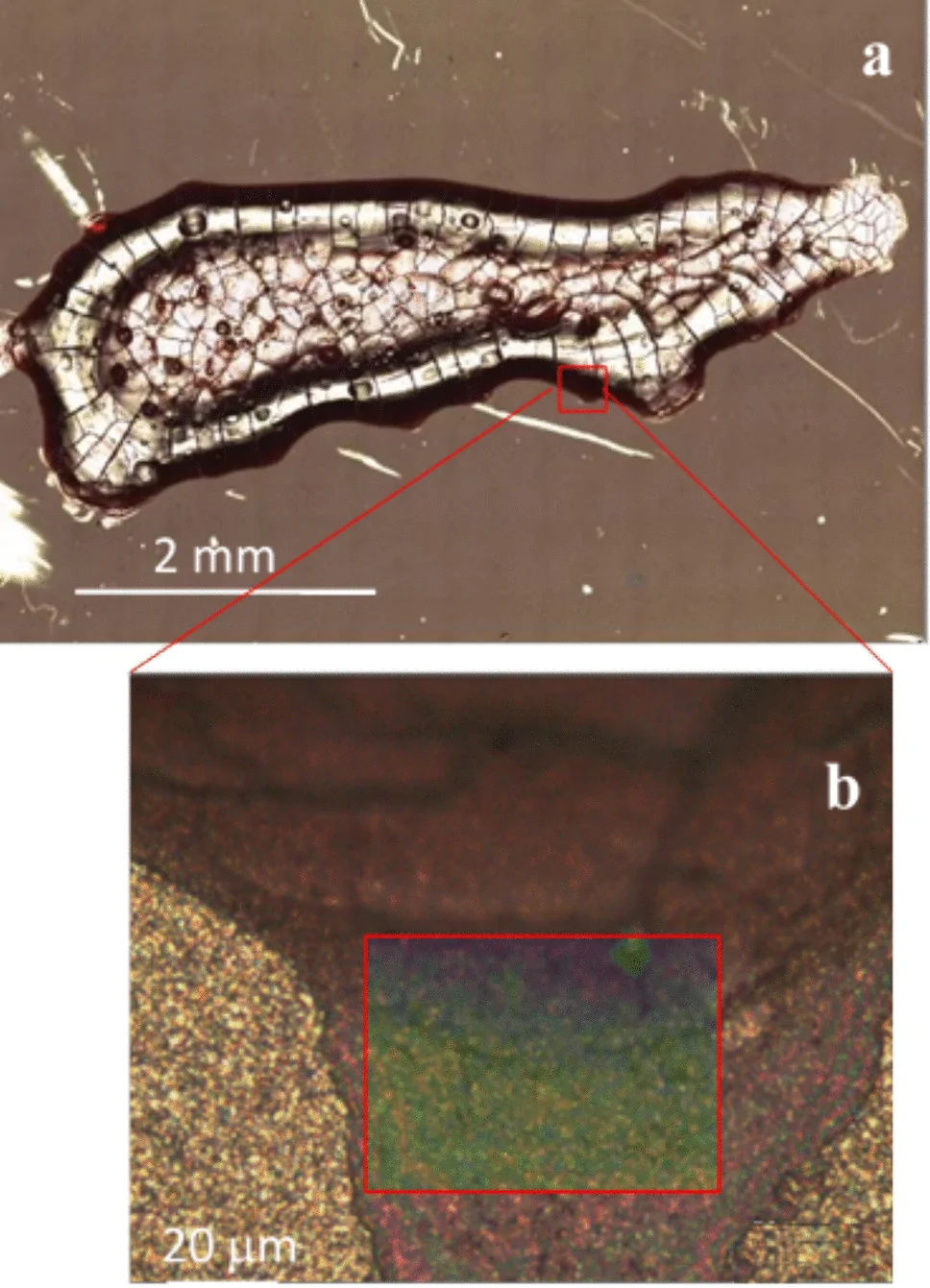
**a**) Optical image of the entire dried drop of whole blood deposited on the surface of the Ag/SiNWs; **b**) Magnification of the border region of the drop showing the spontaneous separation between the solid (dark red, up) and serum (light red, down) parts of the blood sample. The red rectangle delimits the realized Raman map superposed to the optical image: blue and green colours are associated to the different spectral signal coming, respectively, from the solid and the serum regions

An example demonstrating the effectiveness of such spontaneous separation is in Fig. 2, which shows an optical image of the entire dried blood drop (Fig. 2a) and a magnification of the border region, where the solid (dark red, up) and serum (light red, down) areas are clearly distinguishable (Fig. 2b). This physical separation enables the capture of local Raman maps to be used in identifying the unique signal coming from the two sample components. For instance, the red rectangle in Fig. 2b delimits a spectral image realized at the border of the drop and overlaid on the optical image: blue and green colors in the map correspond to the different Raman signal coming from the solid and the serum regions, respectively.

As a consequence, it is possible to obtain the average spectrum over a region of serum enriched with lighter molecules, reducing the large spectral interference due to hemoglobin —which remains mainly sequestrated in the solid part of the drop— and allowing for the identification of other low-concentration biomolecules, such as Hx.

**Fig. 3:**
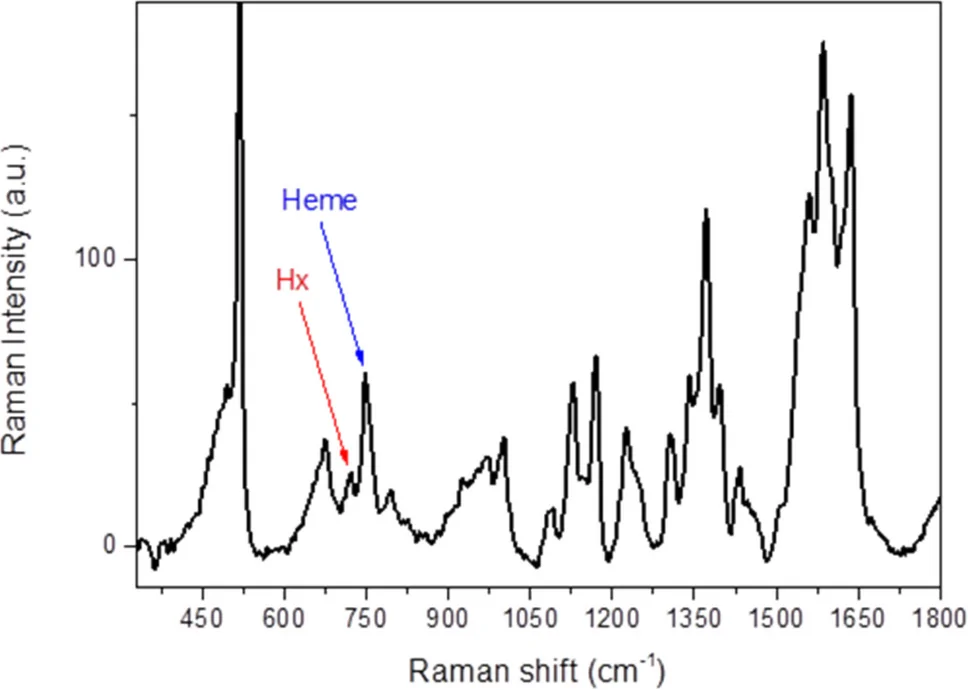
Average Raman spectrum on a map acquired on the serum region at the periphery of the dried blood drop

Figure 3 reports a typical average Raman spectrum calculated on a serum region’s map realized at the periphery of a blood drop. The specific peak ascribed to Hx, located at about 724 cm^−1^, is indicated by the red arrow, while the blue arrow points to the position of the 750 cm^−1^ peak associated with the Heme group of residual hemoglobin [62].

Following previous studies [26, 32], we expect that the intensity of the Hx peak has a specific dependency on the PMI, while the peak at 750 cm^−1^ can be exploited as a reference feature enabling to reduce the influence of minor fluctuations linked to the separation process during blood drop drying and to possible variations in the measurement efficiency (such as those naturally occurring when focusing the excitation laser on a sample with a complex morphology). Thus, the normalization provides the conditions for the comparison of different samples, measured at different times and analysed with different environmental boundary contexts. To verify this assumption, we used the SERS platform to study human whole blood samples from 15 corpses, with known PMI spanning the time window between 3 and 32 h after death. The same quantity of all the liquid samples was deposited on the Ag/SiNWs and left to dry at RT for one hour. Afterwards, identical Raman maps were collected over the serum separated region and averaged to obtain the spectra to be compared. For each average spectrum, the ratio R between the intensities of the identified Hx and Heme bands at 724 and 750 cm^−1^ was then calculated as a PMI marker, after fluorescence and baseline correction.

**Fig. 4:**
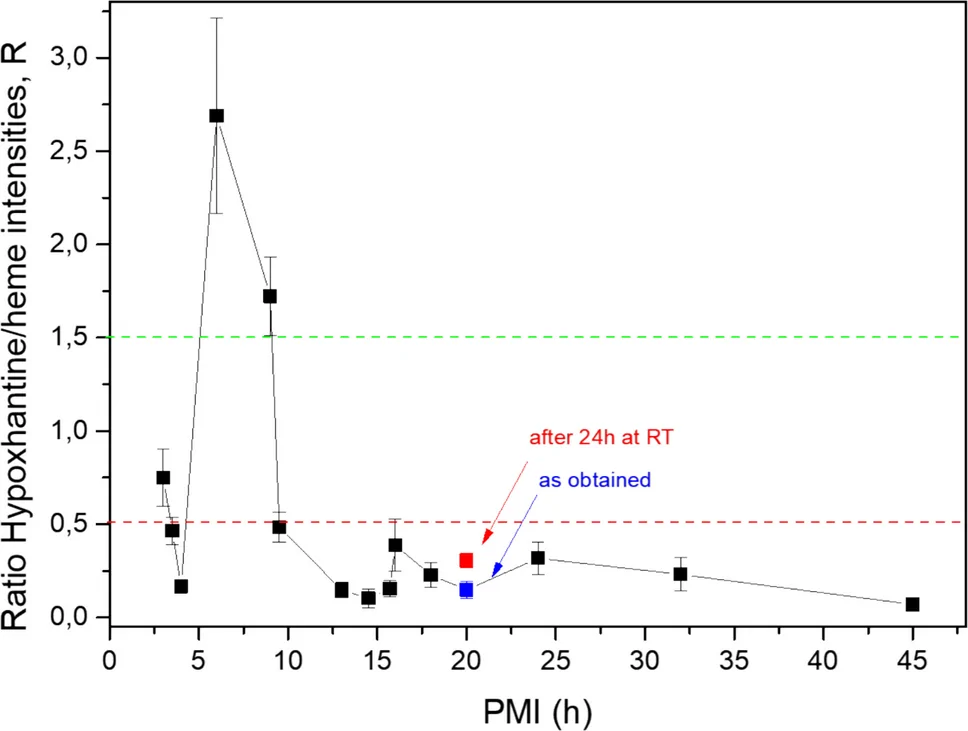
Hx/Heme Raman peak intensity ratio R as a function of PMI. Effects of sample storage at RT are evidenced for the data point corresponding at PMI 20 h

While the intensities of the two peaks are separately reported as Supplementary Data, the values of their ratio are reported in Fig. 4. Quite unexpectedly, they do not confirm a progressive increase of the Hx relative peak intensity with the PMI elapsing, but rather demonstrate a characteristic non-linear behaviour. Within this peculiar trend, which directly depends on the details of the developed measuring protocol, the initial value of R stands at around one, indicating the two peaks have a similar intensity. Then, after an initial decrease, it reaches a maximum around a PMI of 6 h, exceeding the threshold of 1.5, and finally tends to stabilize after about ten hours, remaining below the threshold of 0.5. Thus, three recognizable temporal ranges can be identified: from 3 to 6 h, from 6 to 10 h, and older than 10 h.

**Fig. 5:**
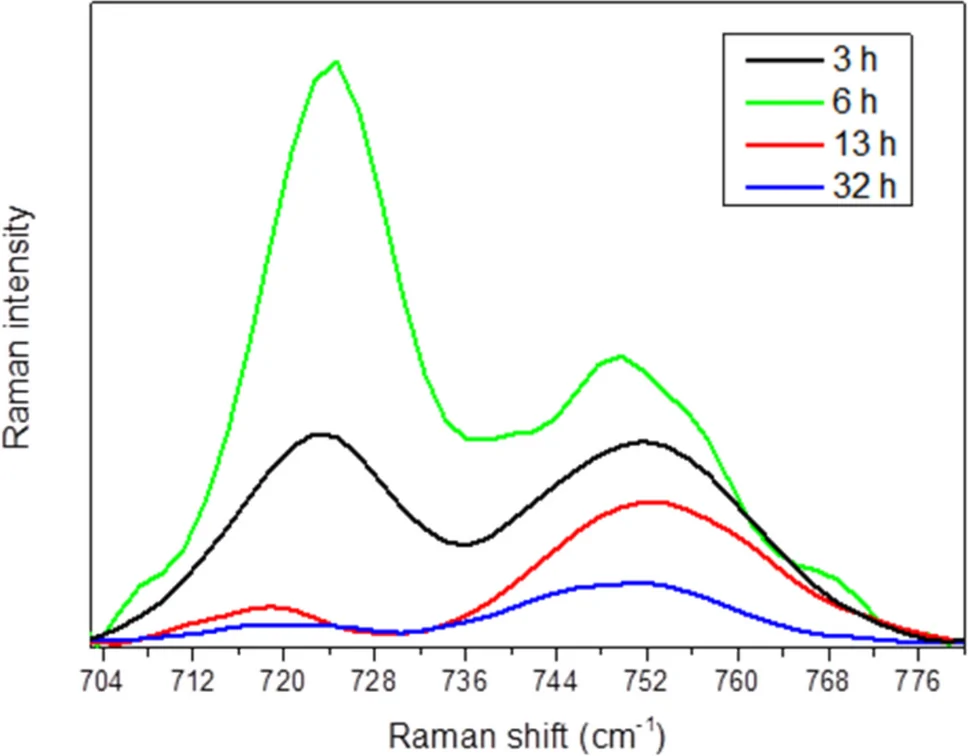
Hx and Heme peaks for average spectra corresponding to PMIs representative of distinguishable temporal ranges

To better appreciate the changing proportion of the two peaks over the three identified temporal ranges, Fig. 5 shows the relevant part of the average spectra for four representative samples corresponding to a PMI of 3 (R = 1), 6 (R > 1.5), 13, and 32 (R < 0.5) hours. As evident from the figure, the overall intensity also starts decreasing after 10 h, so that samples having the same R lower than 0.5, but belonging to the first and third temporal ranges, can be partly distinguished.

The characteristic non-linear behaviour of the Hx relative peak intensity with the PMI elapsing may be explained by considering that, at the cellular level, death is not an instantaneous shutdown. Indeed, when oxygen levels decline, cells switch to anaerobic metabolism and attempts to keep homeostasis intact until they are unable to produce ATP, even by exploiting adenosine salvage pathways. When ATP reserves are exhausted, the rate of degradation towards the final by-products increases rapidly, leading to autolysis and the permanent cessation of their activities. The degradation sequence involves the conversion of ATP to ADP, AMP, IMP, and lastly inosine. Hx is the final link in this catabolic chain, and —as already explained above— the final product of purine catabolism in the bloodstream [48].

Therefore, in the first hours after death, the cellular resiliency probably generates some sort of biochemical latency. Furthermore, until cells undergo lysis through degradation of their structural macromolecules, an intracellular compartmentalization of the purine catabolism products may occur. This phase is possibly reflected in the increasing trend of the ratio R between the Hx and Heme Raman signatures reported in Fig. 4.

Afterwards, when hemolysis becomes predominant, the Hx accumulation in the blood becomes more rapid and consistent, and we can expect a different and more stable behaviour of the ratio R.

Ultimately, at higher PMI, the overall Raman intensity appears to be significantly reduced, as can be appreciated in Fig. 5. Given the incipient tissue putrefaction, this effect can be ascribed to the depletion of Hx precursors, and a conceivable general vibrational structure degradation of the molecules released in the blood and accumulated in the corpse for a longer time. Consequently, the values of R finally decrease towards low and almost constant values.

A full understanding of this non-linear behaviour will require further testing and verification on a larger number of samples. However, it appears clear that our PMI determination method allows to distinguish different phases, highlighting the initial transient imbalance of Hx with respect to Heme by a comparative assessment and reflecting the underlying dynamics of metabolic accumulation in the liquid part of the blood. In particular, the presented data demonstrate that the applied procedure potentially allows for supporting traditional methods for early PMIs estimation, thanks to the fast initial evolution of the Hx contribution’s relative weight in the Raman spectrum of dried serum.

It is important to explicitly point out that this result does not rely on absolute Hx quantification in the blood sample, but rather on Hx spectral appearance in the Raman response of serum, as detected through the described method: what we propose is an approach that exploits a useful parameter (R) and a specific calibration aimed at estimating PMI with reasonable accuracy.

The outlined behaviour can be qualitatively compared with some results reported in the literature using biochemical techniques such as HPLC [25, 28, 32, 36] or Fluorescence Enzymatic Assay [21]. Actually, even if post-mortem increase and accumulation of Hx in mammal biological liquids is generally claimed, the specific data referring to blood serum often present a non-linear and saturating trend after 10 h [28], while very few data are reported for human cadavers, and they are usually influenced and altered by the effects associated with in vitro conservation [62]. In fact, it is known that Hx concentration increases even in whole blood coming from healthy and living donors when it is stored at RT before centrifugation [62]. Hx increases in erythrocyte components due to the endogenous breakdown of AMP within the cells, a process accelerated by the absence of refrigeration. This effect sums up to metabolic modifications due to hypoxia and can impede a correct PMI assessment by standard Hx biochemical quantification.

To highlight the impact of sample conservation for PMI estimation based on our Hx analysis, we repeated the procedure on the same sample after long-term RT lab conservation. As revealed by the data reported in Fig. 4 for blood obtained 20 h after death, while the Hx/Heme intensity ratio for the sample “as obtained” correctly lies below the 0.5 threshold (blue data point), it rises by a factor of 3 after 24 h of tube storage at RT (red data point). Indeed, although in the absence of refrigeration both Hx and hemoglobin molecules are further released due to cell lysis, our method — which is based on Raman analysis of serum enriched with the lighter component — permits evidence of the Hx increase during RT aging in vitro. Moreover, we verified that the vibrational signal after blood RT storage in the tube is little affected, which seems to prove that the molecules released in vitro are less susceptible to metabolic degradation than those accumulated in the corpse for high PMI. This result may be due to the absence of bacteria responsible for the putrefactive processes in the tube, or to their disempowerment mediated by the previous refrigeration of the blood sample.

Therefore, our method demonstrates the ability to follow the presence of Hx in non-treated human corpses’ whole blood samples, allowing the observation of a rapid non-linear evolution of the Hx level within the first hours after death. This notable result is obtained thanks to a fully spectroscopic approach, which eventually provides a supplementary tool for PMI estimation by a label-free, fast, and easy analytical procedure. Additionally, it is potentially applicable to analyse other human biological matrices and is not influenced by parameters such as the density of the sample or impeded by the presence of interfering contaminants like haemoglobin from erythrocytes. Thus, it proves to overcome some evaluation issues reported even for the analysis of anatomically isolated biofluids (such as VH), where the reliability of Hx-related PMI estimates is usually amplified by adopting multivariate regression models or applying exclusion criteria to select only high-quality samples.

Raman spectroscopy is not a new technique, and its use is currently widespread in forensic sciences. Thanks to the large number of peer-reviewed publications, the ability to assess error rates, the wide acceptance of the technique in the scientific community, and the existence of established standard procedures, it satisfies Daubert standards and demonstrated to meet the criteria for scientific evidence admissibility in the U.S. courts [3].

Despite the advantages that would derive from using Raman spectroscopy as a complementary method for evidence analysis in medico-legal inquiries (such as a strong selectivity, reproducibility, ability to analyse any optically accessible sample, not requiring pre-scan preparations, non-destructivity, and potential portability for on-field analysis) [63], this analytical technique is still poorly explored by forensic pathologists, and not commonly used for routine practices or investigations of its specific relevance [64]. Notably, the application of Raman analysis for PMI estimation seems to be limited so far to the field of forensic anthropology [65], to distinguish between forensic and archaeological materials and assess the human skeletal remains' burial time (with examination of time windows up to thousands of years after death or burying).

To the best of our knowledge, the present study is the first to have applied Raman spectroscopy to estimate early PMIs and to have performed this assessment on whole blood from human corpses, thus on samples with real-life post-mortem biochemical modifications [65].

Further work is currently in progress to amplify the number of case studies to corroborate the obtained results and probe possible correlations both with donor characteristics (such as age, cause of death, biological background, lifestyle, medication or drug use, environmental temperature/conditions, gender and so on [66]) and pathological conditions determining hypoxia in life, that certainly affects the post-mortem Hx biochemical pathway. A further study purpose is to extensively appreciate the power of our promising analytical method by analysing larger time windows after death (over 32 h), thus verifying whether Hx shows characterizing behaviors even in medium PMIs.

## Conclusions

We presented a novel approach based on Raman mapping of whole blood, aided by innovative SERS substrates, to rapidly obtain information on the presence of different levels of Hx in serum as a BPM. In this method, only a few microliters of sample are deposited and dried on a disordered array of Ag/SiNWs. The substrate both induces a spontaneous centrifugation effect, separating serum from the cellular component, and enhances the scattering signal from the isolated serum. The ratio R between the vibrational peaks ascribed to Hx, at 724 cm^−1^, and Heme group, at 750 cm^−1^, is then calculated from the average spectrum of the Raman map acquired in the serum region, and it is used as a specific index of the PMI. The procedure was tested on non-treated human whole blood samples from 15 cadavers with known PMI spanning the time window between 3 and 32 h after death, identifying three diverse temporal ranges: from 3 to 6 h, showing decreasing values of R starting from 1; from 6 to 10 h, with high values of R exceeding the threshold of 1.5; and older than 10 h, characterized by a new decrease of R, which stabilizes below the threshold value of 0.5. Our method provides a way to follow the rapid non-linear evolution in the level of Hx in non-treated samples of human corpses’ whole blood in the first hours after death and estimate the PMI in a label-free, fast and simple manner. Moreover, due to the rapidity of the overall procedure and the peculiar properties of the SERS substrate, it is possible to work on whole blood by focusing on the analysis of the serum component and reducing the interference of endogenous increase in Hx concentration associated with erythrocyte damage during sample storage.

This notable result, obtained via a fully optical approach, is compatible with standard and portable spectroscopy and silicon technology, thus offering the basis for prospective on-field applications during medical and forensic site inspections and investigations, while paving the way for future device engineering and automation.

## Supplementary Information

Below is the link to the electronic supplementary material.

Supplementary file1 (DOCX 36 KB)

## Data Availability

The datasets generated and analysed during the current study are available from the corresponding author on reasonable request.
