# Supplementary material for: Raman mapping on Ag-covered nanostructured substrates as an innovative approach for rapid estimation of post-mortem interval on human whole blood
Source: Int J Legal Med. 2026 Jun 26;140(6):3667–76. doi: 10.1007/s00414-026-03882-2 (PMC13658397; doi:10.1007/s00414-026-03882-2)
Supplement: Supplementary file 1 [file 414_2026_3882_MOESM1_ESM.docx]

**SUPPLEMENTARY DATA**

**Raman mapping on Ag-covered nanostructured substrates as an innovative approach for rapid estimation of post-mortem interval on human whole blood**

Sara Sablone^1^, Giuseppe Strisciullo^2^, Andrea Nicola Cardinale^1^, Nicola Antonio Colabufo^3^, Maximilian Ries^4^, Annalisa Convertino^5^, Maurizio Pagliara^6^, Andrea Starace^7^, Valentina Mussi^5^**^*^**

**B**

**A**

**Fig. S1** Raman intensity of Hx (A) and Heme (B) peaks as a function of PMI

While the values of the heme peak do not show a specific trend but just fluctuations to be accounted for, those of Hx are characterized by a phase of initial increase, followed by a settling towards lower values. The normalization of the Hx peak to the heme peak has thus the fundamental technical-operational function of reducing the influence of minor fluctuations linked not only to the separation process during blood drop drying, but also to possible variations in the measurement efficiency, such as those which naturally occur when focusing the excitation laser on a sample with a complex morphology. In other words, the normalization allows us to compare measurements made on different days, with different efficiencies, different environmental boundary conditions, and on samples coming from different cadavers.
